# Supplementary material for: SyNDI: synchronous network data integration framework
Source: BMC Bioinformatics. 2018 Nov 6;19:403. doi: 10.1186/s12859-018-2426-5 (PMC6219086; doi:10.1186/s12859-018-2426-5)
Supplement: Supplementary file 5 — Files of the example presented in Additional file 4. This additional file is a zip package that contains all files used in Additional file 4. Detailed descriptions of each of these files are presented in a README file included in this zip package. (GZ 30411 kb) [file 12859_2018_2426_MOESM5_ESM.gz › SyNDITools/Meme2Fimo/meme_4.9.0_4.tar.gz!/meme_4.9.0/doc/centrimo.html]

CentriMo - localized motif enrichment analysis


# The MEME Suite

## Motif-based sequence analysis tools

### Usage:

`centrimo [options] <sequence file> <motif file>+`

### Description

CentriMo evaluates if motifs are locally enriched in a set of
sequences. By default CentriMo only looks for central enrichment but that
can be changed by supplying the --local option.

### Inputs

#### Motif File

A file containing motifs. Outputs from MEME and DREME are supported
along with minimal MEME format for which there are conversion scripts
avaliable to support other formats. Input motifs that are likely to
appear in the sequences.

#### Sequence File

A file containing FASTA formatted sequences which are expected to
have the most interesing motifs appearing frequently in center.
Typically this will be sequences centered on ChIP-seq peaks.

### Outputs

CentriMo outputs a html file which allows interactive searching and
plotting of motifs which are most centrally enriched. CentriMo also
outputs two text files: centrimo.txt which is a basic tab delimitered
version of the results and site\_counts.txt which lists the count of
motif matches at each offset.

### Options

| Option | Parameter | Description | Default Behaviour |
| --- | --- | --- | --- |
| Input/Output | | | |
| --- | --- | --- | --- |
| --o | name | Create a folder called name and write output files in it. This option is not compatible with -oc as only one output folder is allowed. | The program behaves as if `--oc centrimo_out` had been specified. |
| --oc | name | Create a folder called name but if it already exists allow overwriting the contents. This option is not compatible with -o as only one output folder is allowed. | The program behaves as if `--oc centrimo_out` had been specified. |
| --neg | fasta file | Load the fasta file sequences and scan the motifs against them. The options --disc and --mcc use this comparative sequence set. | The options --disc and --mcc are not avaliable. |
| --bgfile | bg file | Read a zero order background from the specified file. If motif-file is specified then read the background from the motif file. | The program uses the base frequencies in the input sequences. |
| --motif | ID | Select the motif with the ID for scanning. This option may be repeated to select multiple motifs. | The program scans with all the motifs. |
| --motif-pseudo | pseudocount | Apply this pseudocount to the PWMs before scanning. | The program applies a pseudocount of 0.1. |
| --seqlen | length | Use sequences with the length length. | Use sequences with the same length as the first sequence |
| Scanning | | | |
| --- | --- | --- | --- |
| --score | S | The score threshold for PWMs, in bits. Sequences without a match with score ≥ S are ignored. | A score of 5 is used. |
| --optimize\_score |  | Search for the optimal score above the minimum threshold given by the --score option. | The minimum score threshold is used. |
| --maxreg | width | The maximum central region size to consider. | Try all central region sizes up to the sequence width. |
| --norc |  | Do not scan with the reverse complement motif. | Scans with the reverse complement motif. |
| --flip |  | reverse complement matches appear 'reflected' around sequence centers | do not 'flip' the sequence; use rc of motif instead. |
| --local |  | Compute enrichment of all regions. | Compute enrichment of central regions. |
| --disc |  | Use the Fisher exact test to compute enrichment discriminatively. Requires the comparative sequences to be supplied with the --neg option. Use the binomial test to compute enrichment. | |  |
| Output filtering | | | |
| --- | --- | --- | --- |
| --ethresh | thresh | Limit the results to including motifs with better *E*-values. | Include motifs with *E*-values up to 10. |
| Miscellaneous | | | |
| --- | --- | --- | --- |
| --desc | description | Include the text description in the HTML output. | No description in the HTML output. |
| --dfile | desc file | Include the first 500 characters of text from the file desc file in the HTML output. | No description in the HTML output. |
| --noseq |  | Do not store sequence IDs in the output of CentriMo. | CentriMo stores a list of the sequence IDs with matches in the best region for each motif. This can potentially make the file size much larger. |
| -verbosity | 1\|2\|3\|4\|5 | A number that regulates the verbosity level of the output information messages. If set to 1 (quiet) then it will only output error messages whereas the other extreme 5 (dump) outputs lots of mostly useless information. | The verbosity level is set to 2 (normal). |
